# A therapist-focused knowledge translation intervention for improving patient adherence in musculoskeletal physiotherapy practice

**DOI:** 10.1186/s40945-016-0029-x

**Published:** 2017-01-06

**Authors:** Folarin Omoniyi Babatunde, Joy Christine MacDermid, Norma MacIntyre

**Affiliations:** 1 School of Rehabilitation Science, McMaster University, 1400 Main Street West, Hamilton, ON L8S 1C7 Canada; 2 School of Rehabilitation Science, McMaster University, Hamilton, ON Canada; 3 Department of Physical Therapy, University of Western Ontario, London, Canada; 4 Hand and Upper Limb Centre, St Joseph Hospital, London, ON Canada; 5 School of rehabilitation Science, McMaster University, Hamilton, ON Canada

**Keywords:** Adherence, Exercise, Knowledge translation, Active strategy, Physiotherapy

## Abstract

**Background:**

Nonadherence to treatment remains high among patients with musculoskeletal conditions with negative impact on the treatment outcomes, use of personal and cost of care. An active knowledge translation (KT) strategy may be an effective strategy to support practice change. The purpose of this study was to deliver a brief, interactive, multifaceted and targeted KT program to improve physiotherapist knowledge and confidence in performing adherence enhancing activities related to risk, barriers, assessment and interventions.

**Methods:**

We utilised a 2-phase approach in this KT project. Phase 1 involved the development of an adherence tool kit following a synthesis of the literature and an iterative process involving 47 end-users. Clinicians treating patients with musculoskeletal conditions were recruited from two Physiotherapy and Occupational therapy national conferences in Canada. The intervention, based on the acronym SIMPLE TIPS was tested on 51 physiotherapists in phase 2. A pre- and post-repeated measures design was used in Phase 2. Graham’s knowledge-to-action cycle was used as the conceptual framework. Participants completed a pre—intervention assessment, took part in a 1-h educational session and completed a post—intervention assessment. A questionnaire was used to measure knowledge of evidence—based treatment adherence barriers, interventions and measures and confidence to perform evidence—based adherence practice activities. Data was analysed using descriptive statistics (frequency and percentage), Fisher’s exact test and Wilcoxon Sign-Ranked tests.

**Results:**

Barriers and facilitators of adherence were identified under three domains (therapist, patient, health system) in phase 1. Seventy percent of the participants completed the questionnaire. Results indicated that 46.8% of respondents explored barriers including the use of behaviour change strategies and 45.7% reported that they measured adherence but none reported the use of validated outcomes. A significant improvement in post-self-efficacy scores for the four adherence enhancing activities was observed immediately after the workshop.

**Conclusion:**

The use of a multi-modal KT intervention is feasible in an educational setting. A brief interactive educational session was successfully implemented using a toolkit and caused a significant increase in physiotherapists’ knowledge and confidence at performing adherence enhancing activities in the very short-term. Further testing of SIMPLE TIPS on long-term adherence practices could help advance best practices specific to treatment adherence in MSK practice.

**Electronic supplementary material:**

The online version of this article (doi:10.1186/s40945-016-0029-x) contains supplementary material, which is available to authorized users.

## Background

Musculoskeletal (MSK) disorders cause more functional limitations than any other group of disorders within the adult population leading to huge healthcare expenditure and loss of work [1]. Systematic reviews consistently show that exercise is beneficial for key clinical outcomes such as pain, physical function, and quality of life [2, 3]. However, exercise adherence is necessary for program effectiveness, and prevention of recurrent, persistent and disabling problems [4, 5]. Non-adherence to exercise remains high for many MSK conditions [6, 7] and negatively affects treatment effectiveness and duration, personnel use, the therapeutic alliance, waiting times, and cost of care [8, 9]. The WHO defines adherence as “the extent to which a person’s behaviour, corresponds with recommendations from a healthcare professional” (HCP) [10]. This means the associated barriers, intervention and outcome measures (BIM) for improving adherence will vary based on the nature of the treatment recommendations from the HCP. For example, a barrier to home exercise performance could be forgetfulness as compared to transportation difficulties to access clinic-based treatment. Likewise, measuring attendance to treatment sessions may be sufficient to track clinic exercise adherence as compared to assessment of the patient’s unsupervised completion of home exercise. These differences would therefore, inform the type of strategy developed by the HCP in collaboration with the patient to overcome the challenge of nonadherence to treatment.

Evidence shows that to achieve improved patient adherence to treatment, HCPs like physiotherapists need to be supported in efforts to implement a decision-making paradigm that integrates patient preferences, clinical circumstances, personal experience, and scientific evidence for individual patients [11, 12]. This can be achieved through knowledge translation (KT) strategies that optimize how current knowledge can be translated into clinical practice. However, there is growing recognition that problems in knowledge generation rather than KT hinders the knowledge-to-action (KTA) translation process [13]. Effective knowledge transfer has to balance objective knowledge from empirical research such as clinical trials and subjective knowledge such as therapist and patient experience [13, 14]. This bidirectional approach to KT is regarded as a way to overcome some of the obstacles hindering application of available research knowledge in clinical practice [15, 16]. If physiotherapists (PTs) are to play a significant role in promoting patient adherence, further training on MSK treatment adherence management is warranted.

According to the WHO empowering HCPs to manage adherence involves designing context-specific tools adaptable to different settings [10]. Specifically, education and training is required to simultaneously address the areas of knowledge (information on adherence), thinking (the clinical decision-making process) and action (behavioural tools). This suggests that PTs and ultimately patients would benefit from access to specific training and tools for improved treatment adherence and outcomes. Available literature recommends closing the gaps in three key areas: knowledge about instruments for assessing adherence [17, 18], broad determinants and barriers to adherence [12, 19–21] and how to implement evidence-based adherence enhancing interventions [22, 23]. This would increase HCP capacity building for adherence enhancing activities with a potential to increase treatment outcomes in MSK conditions. The purpose of this KT study was two fold:

- Phase 1 – To develop an evidence-based educational strategy to improve therapist knowledge about adherence to treatment and confidence for adherence enhancing activities.
- Phase 2 – To verify its efficacy after a brief interactive educational session. We hypothesised that the median differences between pre-workshop and post-workshop confidence scores would not be equal to zero.

### Theoretical perspective

The knowledge creation component started with the synthesis of the knowledge and previous work in this area. A brief summary of the explicated principles and practices revealed that several theories inform treatment adherence. The main theory chosen for this KT initiative was self-determination theory (SDT) [24, 25], which aims to increase patient’s autonomous motivation and perceived competence to take responsibility for their therapy through the needs supportive environment created by the therapist. SDT has also been integrated with other theories to reduce redundancy between theories and utilise each theory’s strength [26]. For example, autonomous motivation from SDT was shown to be positively associated with attitudes, subjective norms and perceived behavioural control from theory of planned behaviour for MSK injury prevention and rehabilitation [27]. The psychological needs of autonomy and relatedness from SDT also influenced both self-efficacy theory variables: confidence and outcome expectation [28]. Thus, adherence to rehabilitation could plausibly be achieved through strategies that shape the patient’s autonomous motivation by offering support for valued outcomes, providing rehabilitation tasks in an autonomy-supportive manner such as acknowledging commitment, providing rationale and choice, and fostering competence and confidence through clear feedback on effective preventative and rehabilitation exercises and strategies.

### Conceptual framework

**Fig. 1:**
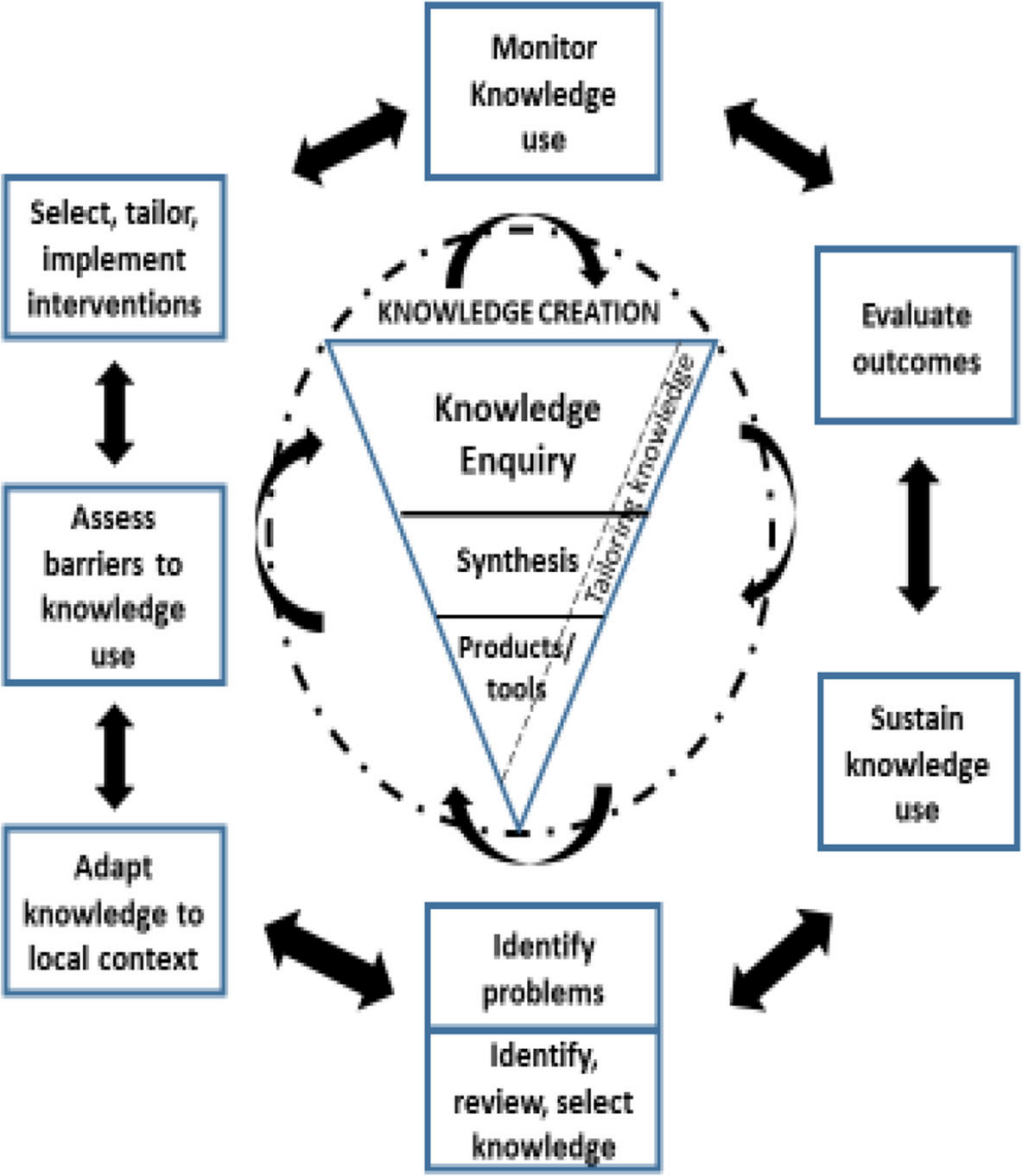
The knowledge-to-action process (Harrison MB, Legare F, Graham ID, Fervers B, Adapting clinical practice guidelines to local context and assessing barriers to their use, 2010;182:E78-E84)

**Fig. 2:**
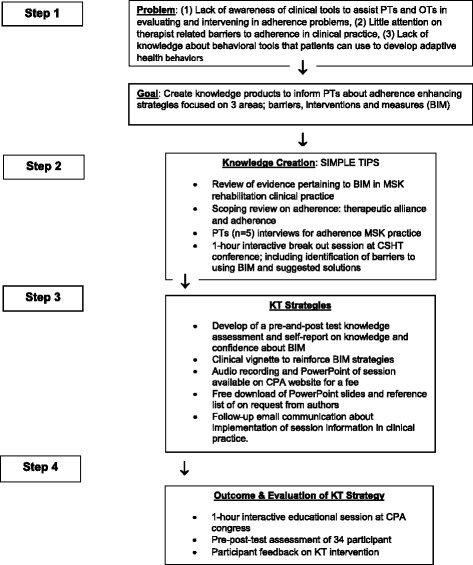
Flow chart of study design based on knowledge translation-to-action cycle: Phase 1 [(Step 1) Identification of MSK practice KT needs, (Step 2) Creating KT intervention], Phase 2 [(Step 3) Implementation of KT Strategy, (Step 4)] KT Evaluation. PT = Physiotherapist, OT = Occupational Therapist, BIM- Barriers-Interventions-Measures, MSK = Musculoskeletal, CSHT = Canadian Society of Hand therapy, CPA = Canadian Physiotherapy Association, KT = Knowledge Translation

The Knowledge-to-Action (KTA) cycle [16] guided our approach to the development and planned implementation of a knowledge resource about adherence BIM. The KTA framework contains two key components: knowledge creation (knowledge funnel) and knowledge action (action cycle). Knowledge creation, where ideas are formulated and techniques and products are developed, lies at the heart of the model as shown in Fig. 1. This process involves knowledge inquiry (primary studies of variable quality addressing a specific question), knowledge synthesis (a summary of the literature using explicit methods), and knowledge tools/products (knowledge synopsis that are presented in a clear and accessible format). Ultimately, it reveals the most refined data that is valid and useful for dissemination. The action cycle describes various activities required for knowledge applications to produce real change as shown in Fig. 1. It consists of seven phases represented by (1) identifying the problem, (2) adapting knowledge to local context, (3) assessing barriers to knowledge use, (4) selecting, tailoring and implementing interventions, (5) monitoring knowledge use, (6) evaluating outcomes and (7) sustaining knowledge use. The relationship between knowledge creation and knowledge action is recognized as fluid and dynamic, each influencing the other. Figure 2 highlights the steps in the KTA cycle used in this study.

### Rationale for KT strategy

We began this KT initiative by identifying the problem. In this case, the problem was a dearth of information to support physiotherapists in MSK practice towards a variety of treatment adherence enhancing goals related to identifying and mitigating barriers, interventions for improving nonadherence and outcome measures for tracking adherence. When we surveyed the literature in planning this study, we found no resource in terms of a decision aid or tool kit available to support clinician’s in tackling the mounting problem of exercise nonadherence reported in MSK rehabilitation practice and research. We had observed anecdotal interest in managing treatment nonadherence in patients with various MSK conditions and valued its potential for increasing treatment outcomes in rehabilitation. The evidence reviewed for this KT intervention through completion of two scoping reviews [Babatunde FO, MacDermid J, MacIntyre N. Characteristics of therapeutic alliance in physiotherapy and occupational therapy for musculoskeletal conditions: a scoping review of the literature; Babatunde FO, MacDermid J, MacIntyre N. Adherence measures for clinic- and home-based exercise in musculoskeletal physiotherapy practice: a scoping review of the literature, unpublished observations] suggests that it would be helpful to create an educational intervention to increase PT’s knowledge and awareness about the problem of nonadherence in MSK physiotherapy. It is anticipated that this approach would facilitate a paradigm shift in PT perspective that supports exploring barriers with patients in detail, tailoring interventions to the needs of the patient and measuring adherence in clinical practice. There has been a relatively low level of knowledge transfer in this area and significant and clinically relevant findings from adherence studies have not been widely implemented in MSK PT practice. Our focus, therefore, was to create a clinically relevant knowledge product to empower therapists about strategies for managing treatment adherence.

### Literature review

#### Evidence on adherence barriers

The WHO Multidimensional Adherence Model proposes that adherence is determined by the interplay of factors related to five constructs; socioeconomic, health care system, condition, treatment, and the patients [10]. Although, the available literature suggests over 200 barriers [10], several barriers to treatment adherence are mainly focused on patient factors with the relative neglect of determinants introduced by HCPs such as PTs [29]. A synthesis of the literature [30] showed strong evidence for the effect of physical (level of physical activity, in-treatment adherence, exercise in previous weeks), psychological (self-efficacy, depression, anxiety/stress, helplessness), socio-demographic (social/family support, barriers to exercise) and clinical (exercise-related pain) barriers in physiotherapy outpatient clinics. Emerging evidence suggests that health care system-related factors such as the patient-therapist relationship (PTR) may be the best predictor of exercise adherence in MSK clinical practice [19]. PTR is partly within the control of the therapist and represents a promising route for promoting exercise adherence in MSK rehabilitation [19]. Therapist-related factors such as tone of voice, positive feedback, empathy, guidance, communication of clear information and trust contributes to improved adherence to exercise [31, 32] and improved treatment outcomes [11]. These factors are potentially modifiable barriers to recovery and require targeted interventions [18]. There was a dearth of literature related to barriers introduced by health care professionals or health organization. In our scoping review, we categorized the different qualities of the therapist and synthesised the evidence on the relationship between adherence and patient-therapist relationship.

#### Evidence on adherence interventions

In view of providing solutions, many adherence interventions directed towards patients have typically focused on education for increased knowledge. Single strategies are less effective for increasing adherence compared to combined cognitive, behavioural, and motivational components [23]. Clearly, interventions to optimize adherence must be extended beyond the provision of advice since information alone is inadequate for creating or maintaining good adherence habits. Although there have been efforts to improve adherence to treatment for patients with MSK conditions, it seems that they have had suboptimal effect due in part to shortcomings in supporting potent behavior change skill sets that can be adopted in clinical practice [9, 33, 34]. In a Cochrane review [23] on interventions for MSK conditions, it was concluded that simple educational and behavioral strategies such as providing feedback or using an exercise contract, providing supervised exercise, follow-up to reinforce exercise behavior, supplementing face-to-face instruction and scheduling convenient treatment times and allowing for rescheduling may enhance adherence. Ultimately, combined interventions may be effective at promoting adherence to exercise. Due to the multidimensional nature of adherence, a broad approach is required to improve the effectiveness of strategies.

#### Evidence on adherence measures

Within clinical practice, objective measures of exercise adherence remain underutilised [17]. Similarly, in randomised controlled trials for MSK disorders, exercise adherence measures are non-existent or limited by use of non-standardized instruments that capture only one domain of adherence [17, 18]. There is currently no gold standard for measuring exercise adherence and more than two hundred measures have been identified in MSK rehab [35]. Only eight measures have been reported to have evidence of psychometric evaluation: Rehabilitation Adherence Questionnaire (RAQ) [36], Community Healthy Activities Model Program for Seniors Activities Questionnaire for Older Adults [37], Hopkins Rehabilitation Engagement Rating scale [38], Adherence to Exercise Scale for Older Patients [39], Sport Injury and Rehabilitation Adherence Scale [40], Pittsburgh Rehabilitation Participation [41], modified rehabilitation Adherence Questionnaire [42], and Rehabilitation Adherence Measure for Athletic Training [43]. In the second scoping review on adherence measures completed by our team, we also identified several measures for adherence. They were categorised as follows: use (clinic-based, home-based) and type of measure (questionnaire, log/diary, Likert-like scale).

## Methods

The professional associations approved the session plan submitted for the two conferences. All participants agreed to allow their data to be analyzed anonymously to develop and assess the effectiveness of the project and signed an informed consent form.

### PHASE 1: Development of SIMPLE TIPS

The process of knowledge product development for this project was initiated by mapping the principles and practices described in the scientific literature on adherence to exercise for MSK conditions with reference to the three topic areas of BIM as follows:

1. Overview of exercise adherence barriers and determinants – Summary of the barriers to adherence and behavioural mechanisms driving adherence related to the patient and the therapist.
2. Interventions for creating or maintaining exercise habits – Summary of behaviour change techniques and tools, choosing best available interventions, developing coping plans, action plans and goal setting.
3. Clinically useful outcome measures – Summary of methods for assessing adherence using home diaries, single-item questionnaires and multi-item questionnaires.

This is important because there is no single strategy deemed effective for managing treatment adherence across all patients, conditions and settings. Consequently, strategies have to be tailored to individual patients [10].

Secondly, we used an iterative process in developing the tool because the information needs to be applicable across a range of MSK practice settings and client population. We understood that the final content and format would emerge through team interactions and feedback from potential users.

Setting: The Canadian Society of Hand Therapy (CSHT) conference held in Montreal, Quebec, Canada in June 2015

Participants: Forty eight participants attended the session and included PTs (*n* = 7) and occupational therapists (OT) (*n* = 41). All participants consented to participate in this KT project. Participants were from various clinical practice settings for adults with MSK conditions. All but four of the participants practiced in Canada. Ten percent ran a private practice.

Session: In order to involve end-users in the development process, we conducted a breakout session titled “Understanding adherence to treatment in hand therapy: focus on barriers, outcome measures and interventions”. The session was targeted at therapists to increase awareness about the areas of BIM. This session was an invited session facilitated by one of the investigators (FB). The 1-h interactive session also sought feedback on three key areas of relevance, accessibility, and format and method that are deemed contributory to the value of KT initiatives for health professionals [44]. Enquiries on practice, patient categories and strategies for identifying barriers to adherence, measuring adherence and managing nonadherence were included during discussion. The session was audio recorded and key themes summarized from the transcripts. Feedback from the first session was used to develop the educational intervention that was used in Phase 2.

The overall challenge we faced in developing a KT tool was how best to present the information to therapists in a useable format that is easily retrievable from memory in clinical practice. Due to the extensive amount of information obtained and necessary for consideration by therapists, we decided to present the information using the mnemonic; “SIMPLE TIPS”. We anticipated that this would facilitate easy uptake of information from a large volume of literature within a short period of time. This was based on a similar approach proposed for managing adherence to medication by Atreja et al. [45] using the word “simple”. We categorised key points from therapist’s feedback and evidence from primary and secondary research based on areas of BIM to develop the educational intervention: SIMPLE TIPS tool (Table 1). This was used in Phase 2 and available on request from the authors.

**Table 1:** KT intervention: SIMPLE TIPS tool kit

Strategy	Key Messages
S – Simplify the regimen	1. Limit exercise prescription to a minimum of 2–5 exercises. 2. Reduce exercises that require special environment and equipment. 3. Match exercises to patient preference, priorities, abilities and prior skills 4. Design programs with as little complexity as possible. 5. Incorporate exercise routine into purposeful dailyactivities.
I – Impart knowledge	1. Talk using nontechnical langauge. 2. Explain the risks and benefits of each treatment option. 3. Create teaching moments using internet information presented by patients. 4. Communicate evidence appropriately to facilitate decision making.
M - Modify psychological response and beliefs	1. Assess and review psychosocial barriers to exercise. 2. Facilitate change behavior by establishing readiness, willingness and confidence for exercise. 3. Include motivational and behavioral adherence enhancement treatment techniques. 4. Avoid talk or action that reinforces pain experience and behavior.
P – Promote therapeutic alliance	1. Create an atmosphere that is both challenging and empowering for patients. 2. Provide constructive feedback about progressor plateau. 3. Seek agreement on treatment goals and tasks. 4. Establish and maintain rapport with patients. 5. Practice patient centred communication.
L - Leave the bias behind	1. Avoid patient stereotypes that connote negative persoanl qualities. 2. Acknowledge and respond to diverse cultural perspectives 3. Make recommendations based on evidence instead of personal beliefs and attitudes. 4. Recognise your own cultural bias and its influence on clinical practice.
E - Evaluate adherence	1. Develop a strategy that patients can use to monitor their own adherence. 2. Review attendance records, exercise skill and overall engagement during clinical encounters. 3. Consider using therapist and patient rated measures to track adherence. 4. Ask simple and direct questions about adherence.
T – Technology can be helpful	1. Use text messaging, mobile phone or email reminders when appropriate. 2. Consider telerehebailitation vis Skype when feasible. 3. Make short exercise videos using patient mobile devices. 4. Include web based treatment tools and outcome measures in the treatment plan.
I – Identify and mitigate barriers	1. Recommend time-efficeint exercises. 2. Provide education on how pain, sleep and energy affects exercise ability. 3. Suggest enjoyable ways to exercise. 4. Plan for transportation and weather challenges. 5. Discuss strategies to help patients remember to exercise.
P – Plan for follow-up	1. Provide booster sessions for long term conditions. 2. Refer patients to community based exercise programs. 3. Maintain updated patient contact information. 4. Dedicate time to reviewing patient progress, pain symptoms and function.
S – Set goals	1. Encourage the setting of SMART goals. 2. Adapt goal setting to the context and the individual. 3. Goal setting should be a collaborative effort that involves other professionals, patients, families and carers. 4. Use patient-specific goal setting measures.

### PHASE 2: Delivering educational intervention and verifying results

Setting: The Canadian Physiotherapy Association annual congress on June, 2015.

Participants: All PTs managing adults with neuromusculoskeletal disorders were targeted for this study. Participants were approached at the door as they entered the venue of the workshop by a graduate student who explained the evaluation component of the session. Consenting respondents were given the questionnaire and instructed to complete the pre-workshop questions before the session started. It was assumed that all participants read and understood the session focus as documented in the conference plan provided to every participant upon registration for the conference.

Session: The educational session was titled “Understanding Adherence to Exercise in Musculoskeletal Physiotherapy; Focus on Barriers, Interventions and Measures led by the principal investigator (FB). The session was included under the theme “Joint and Health” for the mini symposium on Exercise and Physiotherapy for the conference on June 18, 2015. The format included 35 min interactive PowerPoint presentation and 15 min small group discussion using a clinical vignette followed by 10 min of questions and answers. Learning strategies of teaching, discussion, and reflection in practice was used in delivering the session. The session was delivered using principles from problem-based learning and concepts shown to be effective at delivering new information to HCPs based on adapted clinical vignettes [46]. According to Peabody et al. [47, 48] clinical vignettes are a special type of clinical teaching case scenario used to measure professionals’ or trainees’ knowledge and clinical reasoning. They are short descriptions of a person’s health situation which contain precise references to what are thought to be the most important factors in the decision-making process of respondents. Vignettes require respondents to apply their knowledge to a situation, much like they would need to do in a real clinical encounter. This is opposed to choosing from a fixed list of multiple choice options.

There were two aspects to this session: physiotherapist’s knowledge and confidence to apply evidence-based information in the areas of MSK treatment barriers, adherence outcome measures and adherence interventions in their clinical practice. The aim of the session was that participants would be able to do the following by the end of the session: select appropriate outcome measures, describe the various determinants of adherence to exercise, identify evidence based strategies for improving short and long term adherence to exercise, and apply the most up to date literature on exercise adherence to mitigate the problem of nonadherence in MSK rehabilitation clinical practice. The primary outcomes were participant’s knowledge and confidence to apply evidence-based adherence enhancing activities related to barriers, measures and interventions.

A pre-post repeated measures evaluation plan was used. This included a pre-intervention assessment before the session (Time 1) and a post-intervention assessment immediately after the session (Time 2). On the day of the brief interactive session, prior to beginning the intervention (Time 1), the participants were prompted to reflect on their current clinical practice to answer the pre-workshop questions. This was followed by the lecture which ended with discussion of the clinical vignette (Additional file 1). Participants were encouraged to complete the post education questionnaire during the question and answer period or drop complete or incomplete questionnaires on their chairs before exiting the room for their next scheduled educational event. The facilitator encouraged the participants to provide feedback on the format, shared evidence and personal plan to use evidence in practice. The responses were used to write-up a summary of the KT workshop. Attendance and participation feasibility was explored by examining the percentage of those who attended the session, took the questionnaire and returned it.

### Questionnaire

Questionnaire items were designed to identify current and future practices for supporting adherence to exercise during clinical practice. The questionnaire as shown in the appendix (Additional file 2) was divided into two parts; part A was completed before the session started and part B was completed at the end of the session. In Part A, subgroups of items were used to evaluate the personal learning objective, assessment of barriers, measuring exercise adherence, strategies for improving adherence, and confidence to perform adherence enhancing activities. In Part B, subgroups of items were used to evaluate quality of the session, experience at the session, confidence to perform adherence enhancing activities, fulfillment of learning objectives, recommending session to others, significant highlight of session and immediate goal for utilizing knowledge in practice. Items were added to the top of the questionnaire to evaluate respondent demographics, practice characteristics and work setting. The primary source of items were survey tools by Jette et al. [49] and Daudle et al. [50] designed for evaluating physiotherapists behaviour and confidence to implement research evidence in clinical practice. Refer to Additional file 2 for details. The confidence (self-efficacy) to perform adherence enhancing activities was measured (pre- and post) using an 11-point scale that was developed in adherence to guidelines for developing self-efficacy scales [51]. Four items were generated to evaluate self-efficacy to perform each of the following steps of facilitating adherence to exercise according to the WHO [10].: (1) identify patients with potential for poor adherence, (2) assess barriers to exercise, (3) use an outcome measure to assess adherence to exercise, and (4) intervene to improve adherence in practice. To complete the scale, respondents were asked to rate their level of confidence in their ability to perform each activity, using an 11-point scale ranging from 0% (“cannot do at all” to 100% (“certain can do”). Item-level responses were averaged to obtain a summary score ranging from zero to hundred percent.

Six physiotherapists working in education and research (*n* = 2), Neuromusculoskeletal rehabilitation (*n* = 2), orthopaedic outpatients (*n* = 1), private practice (*n* = 1) reviewed the questionnaire and the adherence self-efficacy scale and verified their face validity and relevance. The questionnaire was designed to facilitate completion of each part in 5 to10 min and some questions were re-worded to enhance clarity based on the feedback provided.

## Data Analysis

Descriptive statistics (frequency and percentage) were used to summarize all variables. Preliminary analysis revealed that for the “disagree” and “strongly disagree” categories of the participants experience and the “poor” and “below average” categories of rating of workshop quality, many participants did not input any data. Given the absence of utility data for those categories, subsequent analysis focused on the other categories, namely “average”, “above average”, “excellent” for workshop quality and “strongly agree”, “neutral” “agree” for response on the participant’s experience. The proportion of respondents rating of whether they measure adherence or explore adherence with patients was compared among respondents working across three practice settings (private practice, hospital outpatients/rehabilitation and inpatient/acute care), geographical region (Maritimes, Central, Prairies) and experience (≤10 years, >10 years) with Fisher’s exact due to small sample size and violation of Chi square test assumptions using predicted proportions for expected values. The Wilcoxon sign ranked procedure was used to analyse the difference in confidence between pre- and post-self-efficacy scores. This nonparametric test was selected because the distribution scores were positively skewed and did not meet parametric assumptions. A non-directional test was performed with alpha set at 0.05. All data were managed and analysed using Stata© statistical software (version 13).

## Results

All participants in Phase 1 indicated that adherence to treatment was a big issue in their current practice and they were interested in strategies for managing this problem. The interaction revealed significant knowledge gaps in the areas of BIM for MSK treatment adherence. Only six participants formally assess patient adherence during clinical practice and this involved mostly the use of logs and diaries. Participants were not aware of any valid and reliable instruments for assessing adherence to therapy recommendations. Barriers and facilitators identified during the discussion with therapists are summarized in Table 2. Seven participants were aware of behavioural interventions for adherence and only two reported formal training in this area. Majority of the participants reported that there are few opportunities for professional development in this area of MSK practice and more KT strategies are required to communicate the evidence. The following feedback was provided on the format and method of a feasible clinic focused KT resource: providing access to a downloadable resource through PT and OT organizational websites, webinar or audio recorded presentation between 45–60 min, and development of a MSK rehabilitation focused toolkit and reference list.

**Table 2:** Summary of barriers to and facilitators reported by therapists at the CSHT session

Domain	Barriers	Facilitators
Therapist		
Attitude	• Not therapists’ responsibility • Information overload • Perception of little added value • Resistance to change	• Change perception • Relevant and applicable information • Fostering positive attitude • Knowledge brokers, managers
Competence	• Poor knowledge • Routine practice • Low confidence in own skills	• Education • Reflective practice • Capacity building
Healthcare system		
Colleagues	• Lack of awareness • Varied practice style	• Opportunities through in-service training • Regular meetings and feedback
Outcome Measures	• Poor availability • Difficulty with choice • Feasibility of use	• Increased access • Clear guidelines • Adaptable tools
Practice pattern	• Absence of practice policy • Lack of time	• Create policy • Caseload management, delegation to therapy assistant or Kinesiologist
Patient		
Attitude	• Previous experience • Focus on pain	• Open discussion • Education
Clinic-based treatment	• Transportation difficulties • Cost of care • Lateness to appointments	• Management planning, support • Financial support • Rescheduling
Home-based exercise	• Too many exercises • Early recovery • Forgetting • Lack of time	• Review exercise protocol • Education on healing • Reminders • Action plan, goal setting

### Educational Intervention

Fifty one out of 87 PTs who attended the workshop gave consent and received the questionnaire; 42 were returned out of which 35 questionnaires were completed (70%). Table 3 highlights the participant characteristics. In summary, most respondents were female (57%), had more than 20 years’ experience (28%), and practiced in Nova Scotia (34%). The greatest proportion of respondents worked in facility-based outpatients (25%) and private practice (17%) settings. Less than 15% worked in acute care, rehabilitation or academic/research settings. The least represented setting were school, industrial and animal rehabilitation (<5%). Orthopaedics/MSK was the most therapist specialty (42%). Few therapists (<10%) managed neurological and pediatric conditions while fewer (<5%) managed cardiorespiratory and women health conditions. More than half of respondents (57%) were eager to learn about strategies for improving patient adherence, while 34% were interested in instruments for assessment and identifying barriers. Less than 5% of respondents were interested in application of content of the session in research.

**Table 3:** Characteristics of participants in Phase 2 of the study

Characteristic	Number	Percent
Gender		
Female	20	57.1
Male	15	42.8
Experience		
< 1 year	5	14.2
< 5 years	4	11.4
< 10 years	4	11.4
< 15 years	4	11.4
< 20 years	5	14.2
> 20 years	9	25.7
Geographical area		
Alberta	3	8.5
British Columbia	2	5.7
New Brunswick	3	8.5
New Foundland	1	2.8
Nova Scotia	12	34
Ontario	9	25.7
Quebec	1	2.8
Saskatchewan	2	5.7
Type of Facility		
Acute care hospital	4	11.4
Acute/subacute Rehabilitation	4	11.4
Private outpatient clinic	6	17.1
Facility-based outpatient clinic	9	25.7
Long term care/Community	4	11.4
School system	1	2.8
Industrial	1	2.8
Academic/Research	4	11.4
Animal rehab	1	2.8
Type of condition		
Orthopedic/MSK	15	42.8
Neurological	2	5.7
Cardiorespiratory	1	2.8
Pediatrics	2	5.7
Geriatrics	3	8.5
Women’s Health	1	2.8
Unspecified	6	17.1
No patient care	4	11.4
Non-human	1	2.8

### Barriers to Adherence

Thirty two respondents answered the question about exploring barriers with patients. Almost half (46.8%), reported that they assess barriers, 25% were unsure about assessing adherence and 28% stated that they do not assess barriers to exercises. Among the respondents that answered yes or maybe, 42% (15/25) further highlighted the strategies used to understand barriers to exercise. Only one participant reported the use of an outcome measure; the Tampa scale of Kinesiophobia [52], to capture potential barriers. Three respondents reported the use of the stages of change model [53], two respondent mentioned exploring self-efficacy [54]. Use of open-ended questions on transportation, time issues and home exercise compliance (seven respondents) and monitoring attendance (two respondents) was also reported. Respondents that explore behaviour change techniques were those with more experience (>10 years). The proportion of respondents rating whether they assess barriers by experience, practise setting and region of practice is reported in Table 4. There was significant between-group difference for where clinicians were practicing (*p* = 0.04).

**Table 4:** Proportion of respondents divided by experience, setting and region

Assessment of barriers	Maybe		No		Yes		*p*-value^a^
	*N*	%	*N*	%	*N*	%	
Experience (*n* = 34)							
≤ 10 years	5	63	4	44	10	58	0.526
> 10 years	2	25	5	55	7	42	
Setting (*n* = 31)							
Private practice	3	42	1	12	5	31	0.04
Outpatients	0		1	12	7	43	
Inpatients	4	47	1	12	7	43	
Region (*n* = 30)							
Central	2	29	3	42	5	31	1.000
Maritimes	4	57	3	42	8	50	
Prairies	1	14	1	14	3	18	
Measuring Adherence							
Experience (*n* = 34)							
≤ 15 years	5	63	2	23	12	70	0.080
> 15 years	3	37	7	77	5	29	
Setting (*n* = 32)							
Private practice	3	42	0		7	43	0.035
Outpatients	2	28	3	33	7	43	
Inpatients	2	28	6	66	2	12	
Region (*n* = 31)							
Central	3	37	1	14	6	37	0.213
Maritimes	2	25	5	72	9	47	
Prairies	3	37	1	14	1	6	

^a^Fisher’s exact test

### Measuring treatment adherence

With respect to measuring adherence, 45.7% of the respondents do so routinely while 25.7% and 28.5% were unsure or do not measure adherence respectively. The three most common techniques for measuring adherence were objective change (27.7%), subjective questioning (27.7%) and exercise demonstration and quality (27.7%). Only 11% of respondents utilised attendance records or accurate exercise recall or exercise diaries/logs. Others provided stickers (5.5%) to patients for their calendars. The proportion of respondents rating whether they measure adherence by experience, practise setting and region of practice is reported in Table 3. There was significant between-group difference for where clinicians were practicing (*p* = 0.035). More PTs in facility based inpatient and outpatient practice were more likely to assess adherence compared to those who practice in private practice.

### Reported adherence enhancing strategies

**Fig. 3:**
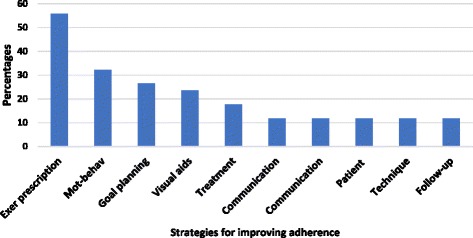
Reported strategies for improving adherence by participants during pre-session assessment of Phase 2

Ten themes emerged from analysis of the response to the question about how participants currently address patient adherence as shown in Table 5 and Fig. 3. Nearly 60% of respondents reported that they manipulate some aspect of exercise prescription to improve adherence. For example, prescribing less than four exercises, incorporating activities into daily routine, prescribing exercises based on patient preference and ensuring proper technique were highly represented. Some forms of education such as ensuring patients understand the rationale for treatment was the most reported strategy and used by 38%. Motivational and behavioural strategies such as behaviour change techniques and focusing on progress were incorporated by 32.3%. Use of technology and visual aids such as reminders and logs and goal planning were each reported by nearly a third of respondents. About a quarter of the respondents utilise visual aids and goal planning. Treatment variables such as supervision or working in groups or with a buddy were included in current practice by 17%. Patient-related interventions and communication each accounted for 11% of current strategies. The least used strategy was function-based outcomes which represented about 5% of responses.

**Table 5:** Strategies used to improve adherence in practice before the educational session

Themes	Components	Percent
Motivational and Behavioral	• Facilitate internal locus of control • Encouragement • Focusing on progress • Focus on long term benefits • Assess stages of change • Graded exposure to exercise • Behavior change techniques	32.3
Communication	• Active listening • Providing feedback	11.7
Exercise prescription	• Limiting number of exercises prescribed • Exercises that are not time consuming • Incorporate exercise into daily activities/routine • Design program around available equipment/resources • Personalized program, patient preferences • Appropriate progression • Prescribe functional and purposeful activity • Fun activities	55.8
Patient	• Leave responsibility to the patient • Identify and understand patient interest • Facilitate active involvement • Group counseling and education	11.7
Education	• Review of anatomical structures and effect of exercise • Understanding of exercise parameters • Improve understanding of rationale for treatment • Patient education • Open discussion • Pain education/fear of re-injury • Education on risk and benefits	38.2
Technique	• Ensure proper demonstration	11.7
Visual aids	• Use of print materials • Attendance sign-in sheets • Use of reminders and logs	23.5
Follow-up	• Review of home exercises regularly • Review after every program progression	11.7
Goal planning and setting	• Plan a daily consistent routine • Build exercise based on goals • Setting meaningful goals • Progress goals • Collaborative goals	26.4
Treatment	• Participation in group program • Use of a workout buddy • Supervised exercise • Simplified programs • Time management	17.6
Technology	• Wearable devices	5.7
Outcomes	• Functional outcomes • Objective performance measures	5.7

### Session impact and quality

**Fig. 4:**
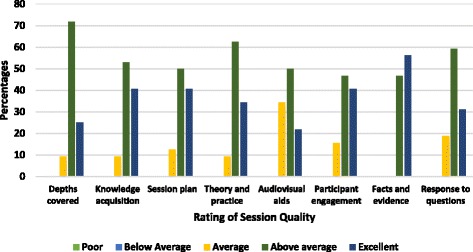
Participants’ rating of the quality of the educational session in Phase 2

**Fig. 5:**
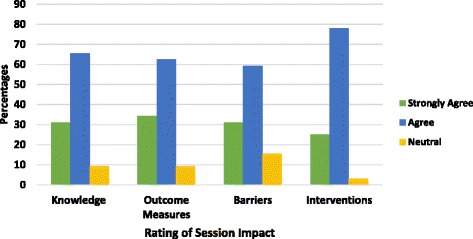
Participants’ rating of Phase 2 session impact on adherence knowledge, measures, barriers and interventions

Most respondents judged six of seven qualities as “above average” and “excellent” as shown in Fig. 4. Most respondents agreed that they advanced their knowledge through participation in the session as shown in Fig. 5. Sixty percent considered the workshop beneficial for advancing their knowledge on importance of assessing adherence. Many respondents (71%) reported that the session was beneficial at increasing awareness of adherence interventions. An increase in knowledge on outcome measures and barriers to adherence was reported by 57% and 54% of participant’s respectively. Less than 10% felt their general insight into importance of adherence and measuring adherence remained unchanged. Approximately 15% reported their level of knowledge about assessing barriers to adherence was unchanged. Very few participants (<5%) were unable to determine if they were better equipped to manage nonadherence. Most participants agreed that the session was impactful for all aspects of the intervention (Fig. 5).

### Self-efficacy for adherence enhancing activities

Participants reported confidence in their own ability to develop and implement strategies to improve adherence (Table 6). A Wilcoxon signed-Ranks test indicated that the post workshop scores were significantly higher than pre workshop scores for adherence risk identification (*z* = 4.42, *p* = .0001), assessing adherence (*z* = 4.45, *p* = .0001), managing adherence (*z* = 4.53, *p* = .0001) and implementing strategies to improve adherence (*z* = 4.66, *p* = .0001).

**Table 6:** Pre and post session self-efficacy scores for adherence enhancing skills

Activity	Average Score (100%)				
Pre-session	Pre-score median (IQR)	Post-score Median (IQR)	Change	Z value	*p*-value^a^
How confident are you in your ability to identity patients at risk of nonadherence	67.5 (30–90)	75 (40–95)	−10	−4.42	0.00001
How confident are you in your ability to assess barriers to exercise	70 (20–90)	80 (35–95)	−10	−4.45	0.00001
How confident are you in your ability to assess whether patients are following treatment recommendations	70 (25–100)	80 (40–100)	−10	−4.532	0.00001
How confident are you in your ability to develop and implement strategies to improve adherence to exercise	60 (25–90)	80 (50–95)	−20	−4.66	0.00001

^a^Wilcoxon Signed Rank test

### Planned Adherence Enhancing Strategy

Twenty-five participants provided an immediate plan of action for the next 2 to 4 weeks based on their experience at the workshop. As shown in Table 7 most participants (52%) reported that they would like to start incorporating some form of objective assessment of adherence in their future clinical practices. Approximately, 32% planned to utilize some of the interventions from knowledge shared into current practice while 28% would seek to improve their communication skills through formal and informal training opportunities. Participants that intended to start considering potential barriers actively with patients or develop a personal learning plan in response to knowledge gained through the session were 16%. One participant’s view remained unchanged with the assertion that all the responsibility still lies with the patient.

**Table 7:** Participant’s plan post session for improving patient adherence

Goals	Details (*n* = 25)	Percent
Outcome measures	Use adherence instruments suggested in practice Review attendance records	52
Barriers	Explore barriers to adherence with patients	16
Interventions	Collaborative and functional goals Use of technology Use patient reminders Assess readiness to change Individualize patient programs	32
Continuous learning	Explore suggested tools for measuring adherence and barriers Review session references Discuss learning objectives with peers Compile a list of outcomes	16
Communication	Motivational interviewing skills Practice active listening Provide feedback Clear information Open ended questions	28
Unchanged	Continue to encourage patient to take responsibility	4

## Discussion

In this article, we outline an example of how an integrated KTA process involving both researchers and clinicians was used to develop and test the effectiveness of a tool kit for translating MSK exercise adherence principles into strategies adaptable in clinical practice. The results of this KT project are very insightful and showed that PTs have a generally positive regard for evidence-based practice for adherence to exercise in MSK rehabilitation. In both sessions, several physiotherapists reported that understanding the concept of adherence is paramount to implementing exercise interventions successfully. The key educational component of this KT strategy was the development of the acronym - “SIMPLE TIPS” from a summary of relevant and highly cited articles from the exercise adherence literature. This approach was well received based on feedback from the participants in both sessions.

In the main KT intervention, respondents showed an increase in confidence in adherence enhancing activities after the session. Overall, the session quality was rated as above average and there was strong agreement that the brief session was impactful. However, very few use objective measures of exercise adherence or explore barriers to exercise with their patients. No respondent was able to identify any validated physiotherapy instruments for assessing exercise adherence used in their clinical practice. Furthermore, most of the strategies suggested for improving patient adherence were patient related. These findings are surprising in view of the recent expansion in the exercise adherence literature and the importance PTs attach to enhancing adherence. Therapists want current research evidence to be presented in simple and adaptable forms that clinicians can implement in practice with minimal additional burden on limited time. Therapists in private practice asserted that change in practice would require tools that do not put additional burden on limited time to deliver care. Similarly, support from managers or practice leaders facilitate adoption of research into public health settings. This findings supports the evidence that clinicians refer to the pressures of the health care environment and administrators’ emphasis on productivity as barriers that directly inhibit their ability to locate, appraise and adapt research evidence into daily clinical practice [55].

This brief KT educational session was designed to bridge the gap between research and clinical practice for adherence to MSK rehabilitation. The goal was to present evidence-based information using an interactive format that both inform and challenge therapists to reflect on current practice and become knowledgeable about strategies for enhancing patient adherence to treatment in their individual practice. To our knowledge, this is the first KT intervention using an active strategy to translate research to clinical practice for exercise adherence among PTs. A recent systematic review of KT strategies in allied health professions by Scott et al. [56] included 11 KT interventions in PT with only two studies [57, 58] reporting a consistent positive effect for the main outcome. One study showed sustained positive attitude and beliefs about evidence based practice was reported in one of the two studies [39]. Most of the studies were focused on the management of MSK conditions or use of clinical practice guideline, used single or multiple intervention, assessed professional/process outcomes and involved participants ranging from 4 to 114 PTs. The authors concluded that the most effective strategy for disseminating of research evidence to allied health professions remains unknown.

In the absence of a consensus on the ways to measure adherence, assess barriers to adherence or enhance adherence in MSK practice, this KT project is a step in the right direction towards communicating available research evidence to clinicians. This then allows clinicians to use their clinical judgement to determine what is best practicable in their individual clinical settings. It is accepted that passive interventions such as postal dissemination do not automatically lead to use in practice or change in practice [59]. Active strategies are therefore preferred. For example, Bekkering et al. [60] showed that an educational session (two 2 h sessions), feedback and use of reminders resulted in a moderate improvement in guideline-consistent behaviour among physiotherapists treating low back pain compared to disseminating guidelines by mail alone. Similarly, Brown et al. [61] utilised a 1 h presentation as the primary strategy among multiple interventions for disseminating information on falls prevention. The authors concluded that the focused nature of the education strategy was responsible for the reported increase in use of fall prevention practice behaviours. These findings infer that there are some benefits to brief educational sessions. To the contrary, a correlation between the length of educational input and likelihood of change in PT practice has been reported [33]. The use of opinion leaders to initiate KT interventions while highly regarded have only had little impact on MSK rehabilitation clinical practice or outcomes [62].

The decision to utilise conferences as an avenue for this KT project was informed by the evidence suggesting that PTs continue to make clinical decisions primarily based on knowledge gained from peers, continuing education conferences, and entry-level education rather than knowledge translated from individually sourced research evidence [59]. PTs are expected to use a decision-making paradigm that combines patient preferences, clinical situations, personal experience, and scientific evidence into an optimal clinical decision for an individual patient [63, 64]. However, little attention has been paid to the best ways in which to support the effort of PTs in the area of adherence to exercise. This is further highlighted by the fact that less than 20% of the respondents had earlier received any type of training to improve understanding and skill development in the three areas relevant to improving adherence practices in musculoskeletal rehabilitation. Ultimately, PTs have to maintain responsibility for their own continuing professional development. Nonetheless, with over 30,000 biomedical journals published each year and estimates that a clinician needs to read 19 articles each day to stay up-to-date [58], it is reasonable to develop more KT initiatives and tools to facilitate clinician access to research evidence.

### Limitations

The nature of this KT outreach and evaluative project does not permit generalization to a larger population. Instead, the focus was on describing the current evidence for managing nonadherence in patients with MSK conditions, the development and implementation of an educational tool aimed at improving therapist knowledge and attitude for a group of PTs. Among the limitations of this project include the lack of validity of the questionnaire used and low reliability of some of the items. The questionnaire used was developed using similar items to those surveyed in the study PTs by Jette et al. [49] and Daudle et al. [50] However, these studies were focused on a general view and barriers to evidence based practice for PTs managing patients with Stroke. Using instruments that are reliable and valid is essential to strengthen the evidence that could be drawn from survey studies. The findings from the analysis could have been skewed by a higher response rate from members of the CPA more interested in adherence and therefore, more positive about it. No effort was made to control for external circumstances that may have impacted the participants’ attitudes, beliefs and behaviors during this project. For example, all the participants may have read the abstract prepared for the session before attending the session. This could have led to a positive regard for and understanding of adherence to exercise. There was also a low number of respondents in this project (87 accessible PTs, 58% gave consent, 42% completed questionnaire). This project was not conducted in a typical setting and we assume that nature of professional conferences where clinicians have to attend multiple sessions in one day may have caused the low response. Finally, the short educational session used may be inadequate to facilitate the substantial behaviour change required to ensure that the evidence communicated is retained and adopted into clinical practice. A direct measure of practice behaviour would have been a preferred way to assess sustained positive effect of the increased confidence to perform adherence enhancing activities among the PTs surveyed.

### Practice implications

The outcome of this KT project has implications for the educational, research and clinical communities. The findings show that therapists in MSK rehabilitation highly regard adherence enhancing activities as contributory to improving patient outcomes but feel inadequate to intervene at various levels. The education community can play a role in providing continuing education at clinical sites and local provincial regions to facilitate adherence enhancing skills in practice who to a great extend include exercise therapy in the management of MSK conditions. Despite the increasing evidence on tools for assessing adherence, this remains a poorly implemented routine in clinical practice. This implies the need to express research information in a way that encourages clinicians to apply evidence in their daily practice. We are currently designing a website to promote access to current evidence on adherence to exercise. It would also be beneficial to partner with local physiotherapy bodies to facilitate the development of an adherence enhancing toolkit since this is believed to promote adoption of consistent behaviour in clinical practice [62]. This could then be made available in various formats including a training manual, website and app to provide a simple stop for clinicians to access published evidence in summary format and potentially overcome barriers of lack of time reported by clinicians [63]. The delivery of educational outreach sessions’ provided at the therapists’ workplace with an actual patient has also been suggested and shown to be effective [63] for facilitating KT and hopefully eventual behaviour change.

## Conclusion

In conclusion, a brief active strategy involving a 1 h interactive educational session was successfully implemented and caused an increase in physiotherapists’ knowledge and confidence at performing adherence enhancing activities. Despite this, it cannot be inferred that this would actually lead to a change in long term adherence behaviour and practices. It is suggested that a fully powered effectiveness study should be conducted to allow for more ongoing support and training in the workplace where the ideas proposed using SIMPLE TIPS can be tested and adapted to patient contexts.

## Additional files

Additional file 1: (DOCX 13 kb)

Additional file 2: Adherence BIM (Barriers, Interventions, Measures) KT Questionnaire. (DOCX 14 kb)

## Abbreviations

BIM: Barriers, Intervention, Measures
CPA: Canadian Physiotherapy Association
CSHT: Canadian Society of Hand Therapy
HCP: Health care professional
KT: Knowledge translation
MSK: Musculoskeletal
OT: Occupational therapist
PT: Physiotherapist
PTR: Patient-therapist relationship
SDT: Self-determination theory
SET: Self-efficacy theory
TPB: Theory of planned behavior
WHO: World Health Organization

## References

[1] Bender JL, Radhakrishnan A, Diorio C, Englesakis M, Jadad AR (2011). Can pain be managed through the internet? a systematic review of randomized controlled trials. Pain.

[2] Walsh NE, Brooks P, Hazes JM, Wals RM, Drenhofer K, Woolf AD, Akesson K, Lidgren L (2008). Bone and joint decade task force for standards of care for acute and chronic musculoskeletal pain: standards of care for acute and chronic musculoskeletal pain: the bone and joint decade (2000–2010). Arch Phys Med Rehabil.

[3] Fuentes JP, Armijo-Olivo S, Magee D, Gross DP (2011). Effects of exercise therapy on endogenous pain-relieving peptides in musculoskeletal pain – a systematic review. Clin J of Pain.

[4] Kay TM, Gross A, Goldsmith C, Santaguida PL, Hoving J, Bronfort G, Cervical Overview Group (2005). Exercises for mechanical neck disorders. Cochrane Database Syst Rev.

[5] Fransen M, McConnell S (2008). Exercises for osteoarthritis of the knee. Cochrane Database Syst Rev.

[6] McLean SM, Burton M, Bradley L, Littlewood C (2010). Interventions for enhancing adherence with physiotherapy: a systematic review. Man Ther.

[7] McLean SM, May S, Klaber MJ, Sharp D, Gardiner E (2007). Prognostic factors for progressive non-specific neck pain. Phys Ther Rev.

[8] Hayden JA, van Tulder MW, Tomlinson G (2005). Systematic reviews: strategies for using exercise therapy to improve outcomes in chronic low back pain. Ann Intern Med.

[9] Kolt GS, McEvoy JF (2003). Adherence to rehabilitation in patients with low back pain. Man Ther.

[10] World Health Organization (WHO) (2003). Adherence to long-term therapies: evidence for action.

[11] Hall AM, Ferreira PH, Maher CG, Latimer J, Ferreira ML (2010). The influence of the therapist-patient relationship on treatment outcome in physical rehabilitation: a systematic review. Phys Ther.

[12] Picorelli AMA, Pereira LSM, Pereira DS (2014). Adherence to exercise programs for older people is influenced by program characteristics and personal factors: a systematic review. J Physiother.

[13] Bowen SJ, Graham ID (2013). From knowledge translation to engaged scholarship: promoting research relevance and utilization. Arch Phys Med Rehabil.

[14] van Twillert S, Postema K, Geertzen JHB, Lettinga AT. Incorporating self-management in prosthetic rehabilitation: case report of an integrated knowledge-to-action process. Phys Ther. 2015;95:640–47. 10.2522/ptj.20130489 24970092

[15] van Twillert S, Geertzen JHB, Hemminga T, Postema K, Lettinga A. Reconsidering evidence-based practice in prosthetic rehabilitation: a shared enterprise. Prosthet Orthot Int. 2013;37:203–11. 10.1177/0309364612459541 23064358

[16] Graham ID, Logan J, Harrison MB, Straus S, Tetroe J, Caswell W, Robinson N (2006). Lost in knowledge translation: time for a map?. J Contin Educ Health Prof.

[17] Bollen JC, Den SG, Sieget RJ, Howe TE, Goodwin VA (2014). A systematic review of measures of self-reported adherence to unsupervised home-based rehabilitation exercise programmes, and their psychometric properties. BMJ Open.

[18] Hall AM, Kamper SJ, Hernon M, Hughes K, Kelly G, Lonsdale C, Hurley DA, Ostelo R (2015). Measurement tools for adherence to non-pharmacologic self-management treatment for chronic musculoskeletal conditions: a systematic review. Arch Phys Med Rehabil.

[19] Wright BJ, Galtieri NJ, Fell M (2014). Non-adherence to prescribed home rehabilitation exercises for musculoskeletal injuries: the role of the patient-practitioner relationship. J Rehabil Med.

[20] Bennell LK, Dobson F, Hinman RS. Exercise in osteoarthrits: moving from prescription to adherence. Best Pract Res Clin Rheumatol. 2014;28:93-117. 10.1016/j.berh.2014.01.009 24792947

[21] Beinart NA, Goodchild CE, Weinman JA, Ayis S, Godfrey EL (2013). Individual and intervention-related factors associated with adherence to home exercise in chronic low back pain: a systematic review. Spine J.

[22] Aitken D, Buchbinder R, Jones G, Winzenberg T (2015). Interventions to improve adherence to exercise for chronic musculoskeletal pain in adults. Aust Fam Physician.

[23] Jordan JL, Holden MA, Mason EEJ, Foster NE (2010). Interventions to improve adherence to exercise for chronic musculoskeletal pain in adults. Cochrane Database Syst Rev.

[24] Ng YY, Ntoumanis N, Thogersen-Ntoumani C, Deci EL, Ryan RM, Duda JL, Williams GC (2012). Self-determination theory applied to health contexts. Perspect Psychol Sci.

[25] Murray A, Hall AM, Williams GC, McDonough SM, Ntoumanis N, Taylor IM, Jackson B, Matthews J, Hurley DA, Lonsdale C (2015). Effect of self-determination theory-based communication skills training program on physiotherapists’ psychological support for their patients with chronic low back pain: a randomized controlled trial. Arch Phys Med Rehabil.

[26] Noar SM, Zimmerman RS (2005). Health behavior theory and cumulative knowledge regarding health behaviors: Are we moving in the right direction?. Health Educ Res.

[27] Chan DKC, Hagger MS (2012). Self-determined forms of motivation predict sport injury prevention and rehabilitation intentions. J Sci Med Sport.

[28] Sweet SN, Ms F, Strachan SM, Blanchard CM (2012). Testing and integrating self-determined theory and self-efficacy theory in a physical activity context. Can Psychol.

[29] Salbach NM, Jaglal SB, Korner-Bitensky N, Rappolt S, Davis D (2007). Practitioner and organizational barriers to evidence-based practice of physical therapists for people with stroke. Phys Ther.

[30] Jack K, McLean SM, Moffett JK, Gardiner E (2010). Barriers to treatment adherence in physiotherapy outpatient clinics: a systematic review. Man Ther.

[31] O’Keeffe M, Cullinane P, Hurley J, Leahy I, Bunzil S, O’sullivan PB, O’sullivan K (2016). What infleunces patient-therapist interactions in musculoskeletal physical therapy? qualitative systematic review and meta-synthesis. Phys Ther.

[32] Hinman RS, Delany CM, Campbell PK, Gale J, Bennell KL (2016). Physical therapists, telephone coaches and patients with knee osteoarthritis: qualitative study about working together to promote exercise adherence. Phys Ther.

[33] Gray H, Howe T (2013). Physiotherapists’ assessment and management of psychosocial factors (yellow and blue flags) in individuals with back pain. Phys Ther Rev.

[34] Synott A, O’Keeffe M, Bunzli S, Dankaerts W, O’Sullivan P, O’Sullivan K (2015). Physiotherapists may stigmatise or feel unprepared to treat people with low back pain and psychosocial factors that influence recovery: a systematic review. J Physiother.

[35] McLean SM, Holden M, Haywood K, Potia T, Gee M, Mallett R, Bhanbro S (2015). Exercise adherence measures – why we need to start again. Findings of a systematic review and consensus workshop. Physiotherapy.

[36] Brewer BW, Daly JM, van Raalte JL, Petipas AJ (1999). A psychometric evaluation of the rehabilitation adherence questionnaire. J Sport Exer Psychol.

[37] Stewart AL, Mills KM, King AC, Haskell WL, Gillis D, Ritter P (2001). CHAMPS physical activity questionnaire for older adults: outcomes for interventions. Med Sci Sports Exer.

[38] Lenze EJ, Munin MC, Quear T, Dew MA, Rogers JC, Begley AE, Reynolds CF (2004). The Pittsburgh rehabilitation scale: reliability and validity of a clinician-rated measure of participation in acute rehabilitation. Arch Phys Med Rehabil.

[39] Hardage J, Peel C, Morris D, Graham C, Brown C, Foushee HR, Braswell J (2007). Adherence to exercise scale for older patients (AESOP): a measure for predicting exercise adherence in older adults after discharge from home health physical therapy. J Geriatr Phys Ther.

[40] Kolt GS, Brewer BW, Pizzarri T, Schoo AMM, Garrett N (2007). The sport injury rehabilitation adherence scale: a reliable scale for use in clinical physiotherapy. Physiotherapy.

[41] Kortte KB, Falk LD, Castillo RC, Johnson-Greene D, Wegener ST (2007). The Hopkins rehabilitation engagement rating scale: development and psychometric properties. Arch Phys Med Rehabil.

[42] Shin JT, Park R, Song WI, Kim SH, Kwon SM (2010). The redevelopment and validation of the rehabilitation adherence questionnaire for injured athletes. Int J Rehabil Res.

[43] Granquist MD, Gill DL, Appaneal RN (2010). Development of a measure of rehabilitation adherence for athletic training. J Sport Rehabil.

[44] Pentland D, Forsyth K, Maciver D, Walsh M, Murray R, Irvine L, Sikora S (2011). Key characteristics of knowledge transfers and exchange in healthcare: integrative literature review. J Adv Nurs.

[45] Atreja A, Bellam N, Levy SR (2005). Strategies to enhance patient adherence: making it simple. MedGenMed.

[46] Learman KE, Ellis AR, Goode AP, Showalter C, Cook CE (2014). Physical Therapists’ clinical knowledge of multidisciplinary low back pain treatment guidelines. Phys Ther.

[47] Peabody J, Luck J, Glassman P, Dresselhaus T, Lee M (2000). Comparison of vignettes, standardized patients, and chart abstraction: a prospective validation study of three methods for measuring quality. JAMA.

[48] Peabody J, Luck J, Glassman P, Jain S, Hansen J, Spell M (2004). Measuring the quality of physician practice by using clinical vignettes: a prospective validation study. Ann Intern Med.

[49] Jette DU, Bacon K, Batty C (2003). Evidence-based practice: beliefs, attitudes, knowledge and behaviours of physical therapists. Phys Ther.

[50] Daudle E, Faus MJ, Santonja FJ, Fernandez-Limos F (2009). Effectiveness of a videoconference training course on implementing pharmacy services. Pharm Word Sci.

[51] Bandura A (2006). Guide for constructing self-efficacy scales.

[52] Miller RP, Kori S, Todd D (1991). The Tampa Scale: a measure of kinesiophobia. Clin J Pain.

[53] Prochaska JO, DiClemente CC, Norcross JC (1992). In search of how people change: applications to addictive behaviours. Am Psychol.

[54] Bandura A (1993). Perceived self-efficacy in cognitive development and functioning. Educ Psychol.

[55] Gross DP, Lowe A (2009). Evaluation of a knowledge translation initiative for physical therapists treating patients with work disability. Disabil Rehabil.

[56] Scott SD, Albrecht L, O’Leary K, Ball GDC, Hartling L, Hofmeyer A, Jones CA, Klassen TP, Kovacs Burns K, Newton AS, Thompson D, Dryden DM (2012). Systematic review of knowledge translation strategies in the allied health professions. Implement Sci.

[57] Klassen T, Jadad A, Moher D (1998). Guides for reading and interpreting systematic reviews. Arch Ped Adol Med.

[58] Schreiber J, Stern P, Marchetti G, Provident I (2009). Strategies to promote evidence-based practice in paediatric physical therapy: a formative evaluation pilot project. Phys Ther.

[59] O’Brien M (2001). Keeping up to date: continuing education improvement strategies and evidence-based physiotherapy practice. Physiol Theory Pract.

[60] Bekkering GE, Hendriks HJM, van Tulder MW, Hoejenbos M, Oostendorp RAB, Bouter LM (2005). Effect of the process of care of an active strategy to implement clinical guidelines on physiotherapy for low back pain: a cluster randomised controlled trial. Qual Safe Health Care.

[61] Brown CJ, Gottschalk M, Van Ness PH, Fortinsky RH, Tinetti ME (2005). Changes in physical therapy providers’ use of fall prevention strategies following a multicomponent behavioural change intervention. Phys Ther.

[62] Mikhail C, Korner-Bitensky N, Rossignol M, Dumas JP (2005). Physical therapists’ use of interventions with high evidence of effectiveness in the management of a hypothetical typical patient with acute low back pain. Phys Ther.

[63] Langley C, Faulkner A, Watkins C, Gray S, Harvey I (1998). Use of guidelines in primary care – Practitioners’ perspectives. Fam Pract.

[64] Overmeer T, Boersma K, Denison E, Linton S (2011). Does teaching physical therapists to deliver a biopsychosocial treatment program result in better patient outcomes? a randomized controlled trial. Phys Ther.

