# Supplementary material for: A therapist-focused knowledge translation intervention for improving patient adherence in musculoskeletal physiotherapy practice
Source: Arch Physiother. 2017 Jan 6;7:1. doi: 10.1186/s40945-016-0029-x (PMC5759916; doi:10.1186/s40945-016-0029-x)
Supplement: Supplementary file 1 — (DOCX 13 kb) [file 40945_2016_29_MOESM1_ESM.docx]

**Clinical vignette**

You are a licensed physiotherapist (PT) with 10 years of clinical experience. You practice with 2 other PTs and 2 occupational therapists in a publicly funded outpatient orthopedic clinic. Other staff members include a receptionist/ofﬁce manager and 2 PT/OT Assistants.

During your initial evaluation, you examine a 56-year-old man who complains of bilateral low back and buttock pain. He arrives via direct access. His wife, who you recently treated, convinced her husband to see you because his symptoms have now lasted longer than 3 weeks. During the patient history, he indicates that he has a long-term history of low back and buttock pain that is worsened during walking, lessened during sitting, and subsides during the evening once his activity level declines. He indicates that he takes no medication at present and has not received any therapeutic care within the previous 6 weeks. He does indicate that his symptoms are cyclical and that a similar problem has plagued him over the last 5 years, intermittently, but usually resolve within 2 to 3 weeks. During his span of 5 years with the condition, he has never received any imaging. He reports no pain below the buttock, no history of cancer, and not having any diagnoses associated with rheumatoid arthritis, ankylosing spondylitis, or other forms of spinal arthropathies. He reports that because his pain declines in the evening with less activity, his self-treatment has consisted of rest and diminished movement. The clinical examination yields reproduction of symptoms during repeated lumbar side ﬂexion to the right. No sensory or motor deﬁcits were found. Lumbar spine range of motion is limited during all movements.

You recommended 6 treatment sessions and a course of home exercise program. On your review after 4 treatments you notice that the patient has not made considerable progress on physical and functional outcomes. He comes late of appointments and always eager to go home because he wants to avoid traffic. You are concerned that he does not do much at home and want to explore this further.

Refection

1. What are the patient and therapist related factors that could limit participation in exercise.
2. What are some of the barriers to this patient participating in self-directed exercise?
3. How would you assess exercise adherence at home and in the clinical for this client?
4. If you discover that this patient has poor adherence to exercise, what strategies would you use to address nonadherence?
