# Supplementary material for: A therapist-focused knowledge translation intervention for improving patient adherence in musculoskeletal physiotherapy practice
Source: Arch Physiother. 2017 Jan 6;7:1. doi: 10.1186/s40945-016-0029-x (PMC5759916; doi:10.1186/s40945-016-0029-x)
Supplement: Supplementary file 2 — Adherence BIM (Barriers, Interventions, Measures) KT Questionnaire. (DOCX 14 kb) [file 40945_2016_29_MOESM2_ESM.docx]

**Additional Material 2:** Adherence BIM (Barriers, Interventions, Measures) KT Questionnaire

| Demographics |  |
| --- | --- |
| Area of practice or research (e.g. adult/MSK): | |
| Setting (e.g. outpatients): | |
| Location of practice: | |
| Experience (years): | |
| Email (optional): | |
|  | |
| Pre-workshop Reflection | |
| 1. What is your learning objective for attending this session? | |
| Response: | |
| 2. Do you assess barriers to exercise adherence in your practice: No [ ] Yes [ ] Maybe [ ] | |
| If you answered yes/maybe, please explain: | |
| 3. Do you measure adherence to exercise in your practice: No [ ] Yes [ ] Maybe [ ] | |
| If you answered yes/maybe, please explain: | |
| 4. Name 3 strategies you currently use to improve adherence to treatment  a.  b.  c. | |
| 5. *For each of the following activities, please indicate how conﬁdent you are in your current level of ability:*  0% 10% 20% 30% 40% 50% 60% 70% 80% 90% 100%  Cannot Moderately Certain  Do at All Certain Can Do Can Do | |
| How confident are you in your ability to:  1……identify patients at risk of nonadherence in practice? _____%  2……assess barriers to exercise in your practice? _____%  3……accurately assess whether patients are following treatment recommendations? _____%  4……develop and implement strategies to improve patient adherence to exercise? _____% | |
| Comments/notes: | |

| Post Workshop Feedback | | | | | |
| --- | --- | --- | --- | --- | --- |
| 6. Please, rate the quality of this session | | | | | |
| Session characteristics | **Poor** | **Below average** | **Average** | **Above average** | **Excellent** |
| 6.1 Depth of aspects covered |  |  |  |  |  |
| 6.2 Knowledge acquisition |  |  |  |  |  |
| 6.3 Planning of session |  |  |  |  |  |
| 6.4 Proportion of theory/practice |  |  |  |  |  |
| 6.5 PowerPoint slides |  |  |  |  |  |
| 6.6 Participant engagement |  |  |  |  |  |
| 6.7 Communication of facts/evidence |  |  |  |  |  |
| 6.8 Response to questions |  |  |  |  |  |
| 7. Please answer the following questions regarding your experience at this workshop | | | | | |
| Workshop Outcomes | **Strongly agree** | **Disagree** | **Neutral** | **Agree** | **Strongly**  **Agree** |
| 7.1 I advanced my knowledge of exercise adherence |  |  |  |  |  |
| 7.2 I learned how to measure exercise adherence |  |  |  |  |  |
| 7.3 I learned how to assess barriers to adherence |  |  |  |  |  |
| 8. *For each of the following activities,* *please indicate how conﬁdent you are in your current level of ability after the session:*  0% 10% 20% 30% 40% 50% 60% 70% 80% 90% 100%  Cannot Moderately Certain  Do at All Certain Can Do Can Do | | | | | |
| After attending this workshop how confident are you in your ability to:  8.1……identify patients at risk of nonadherence in practice? _____%  8.2……assess barriers to exercise in your practice? _____%  8.3……accurately assess whether patients are following treatment recommendations? _____%  8.4……develop and implement strategies to improve patient adherence? _____% | | | | | |
| 9. Part 1: Did this session fulfill your reason for attending?  Yes, absolutely [ ], ­­­­­­­­­­­­­ Yes, but not to my full extent [ ], No [ ] | | | | | |
| 10. Would you recommend this session for a colleague?  Yes [ ], Maybe [ ], No [ ] | | | | | |
| 11: What was the most beneficial aspect of this workshop? | | | | | |
| 12. Part 8: What do you plan to do differently in your practice going forward (2-4 weeks)? | | | | | |
